# The effect of metabolic syndrome on controlled ovarian stimulation outcome in infertile women with polycystic ovary syndrome undergoing assisted reproductive technology cycles

**DOI:** 10.20945/2359-3997000000518

**Published:** 2022-09-20

**Authors:** Ashraf Moini, Tawoos Rezaee, Ashraf Aleyasin, Arezoo Arabipoor, Marzieh Eslami Moayed

**Affiliations:** 1 Tehran University of Medical Sciences Arash Women’s Hospital Department of Obstetrics and Gynecology Tehran Iran Department of Obstetrics and Gynecology, Arash Women’s Hospital, Tehran University of Medical Sciences, Tehran, Iran; 2 Tehran University of Medical Sciences Breast Disease Research Center (BDRC) Tehran Iran Breast Disease Research Center (BDRC), Tehran University of Medical Sciences, Tehran, Iran; 3 ACECR Royan Institute for Reproductive Biomedicine Reproductive Biomedicine Research Center Tehran Iran Department of Endocrinology and Female Infertility, Reproductive Biomedicine Research Center, Royan Institute for Reproductive Biomedicine, ACECR, Tehran, Iran; 4 Tehran University of Medical Sciences Arash Women’s Hospital Infertility Ward Tehran Iran Infertility Ward, Arash Women’s Hospital, Tehran University of Medical Sciences, Tehran, Iran; 5 Tehran University of Medical Sciences Shariati Hospital Department of Infertility Tehran Iran Department of Infertility, Shariati Hospital, Tehran University of Medical Sciences, Tehran, Iran; 6 Tehran University of Medical Sciences Shariati Hospital Department of Obstetrics and Gynecology Tehran Iran Department of Obstetrics and Gynecology, Shariati Hospital, Tehran University of Medical Sciences, Tehran, Iran

**Keywords:** Metabolic syndrome, polycystic ovary syndrome, assisted reproductive technology, pregnancy outcome

## Abstract

**Objective:**

To evaluate the effect of metabolic syndrome (MetS) diagnosis on oocyte quality and pregnancy outcomes in infertile women with polycystic ovary syndrome (PCOS) who undergoing antagonist-controlled ovarian stimulation (COS) and in vitro fertilization/intracytoplasmic sperm injection (IVF/ICSI) cycles.

**Subject and methods:**

This prospective cohort study was conducted from November 2019 to November 2020 across two university-affiliated infertility centers in Iran. The PCOS diagnosis was defined according to the Rotterdam criteria. The patients prior to IVF/ICSI cycles were evaluated for MetS diagnosis. MetS was detected according to the National Cholesterol Education Program/Adult Treatment Panel III with the presence of at least three or more of the specific clinical criteria. The cycle outcomes were compared between MetS and non-MetS groups.

**Results:**

Overall, 68 eligible infertile PCOS patients with MetS diagnosis and 126 without MetS participated. The MetS diagnosis was associated with the increased requirement of gonadotropins and the COS duration significantly (P = 0.001). Although the total numbers of retrieved and MII oocytes, obtained and top-quality embryos as well as clinical pregnancy and live birth rates in the MetS group were lower than those of in the non-MetS group, the differences were not statistically significant (P > 0.05). In follow-up of the obstetrics complications, the rate of preeclampsia was significantly higher in patients with MetS (P = 0.02).

**Conclusion:**

MetS diagnosis in PCOS patients was associated with non-significant poor COS and pregnancy outcome. Further studies with larger sample sizes are recommended to clarify the risk of MetS in patients undergoing ART cycles.

## INTRODUCTION

Polycystic ovary syndrome (PCOS) is an endocrinopathy with heterogeneous manifestations that affects up to 6%-21% of reproductive-age women and is a challenging factor of female infertility in assisted reproduction technology (ART) cycles (1,2). The prevalence rates of PCOS and its phenotypes are mainly related to the environmental, cultural, and genetic factors, as well as the diagnostic criteria (3).

Infertility is one of the main problems of PCOS patients that has been reported in 40% of them. Infertility management options in PCO women include ovulation stimulation, intrauterine insemination, and assisted reproductive techniques (ART) cycles. Although PCOS women present high ovarian response during the controlled ovarian stimulation (COS) process and it might be associated with decreased oocyte quality (4) and also low fertilization rate (5), high rates of cycle cancelation (5), and miscarriage are reported for these women (6). It has been reported that disorders in oocyte maturation and development of embryos in PCOS women may be related to endocrine/paracrine factors, metabolic dysfunction, and changes in the intracellular microscopic environment during folliculogenesis and follicular maturation (7).

Several studies demonstrated that PCOS women have a higher risk of developing metabolic disorders which are related to obesity and metabolic features but not associated with indices of hyperandrogenism (8). Evidence-based guidelines in PCOS recommend screening for features of metabolic syndrome (MetS) (9). MetS is an endocrine disorder characterized by cardiovascular risk factors such as insulin resistance (IR), abdominal obesity, inflammation, hypertension, and dyslipidemia, which is now becoming an epidemic (10). One in four women in the United States is at risk of MetS, and its incidence is developing every year (10). The complications of MetS have overlaps with obesity and PCOS which are linked to infertility and poor reproductive outcome. Therefore, the relationship between metabolic syndrome and reproductive dysfunction is a debating issue for study (10).

To the best of our knowledge, few studies have evaluated the effect of MetS on oocyte quality or pregnancy outcomes in women with PCOS who underwent in vitro fertilization (IVF). Some studies have reported that poor oocyte and embryo quality and fertility outcomes in PCOS patients undergoing IVF may be due to changes in the microenvironment of the follicular fluid due to metabolic changes (11,12). Similarly, He and cols. showed an association between MetS and poor pregnancy outcomes in women with PCOS who underwent IVF. However, the authors mentioned that the results were unpowered caused by a lack of evaluation of confounding factors (13). Since studies in this field are inadequate that no definitive conclusions can be drawn from them; consequently, this study was designed to determine the effect of metabolic syndrome on oocyte quality and pregnancy outcomes in infertile women with PCOS who underwent antagonist-controlled ovarian stimulation and IVF cycles.

## SUBJECT AND METHODS

This prospective study was conducted from November 2019 to November 2020 across two university-affiliated infertility centers in Iran. The study protocol was approved by the scientific board and the ethics committees of the Tehran University of Medical Sciences (approval code: IR.TUMS.MEDICINE.RE.1399.010). The study’s aim was explained to the patients who meet the inclusion criteria and written consent was obtained from the participants.

All women with PCOS diagnosis who were referred to Shariati Hospital and Arash Women’s Hospital for in vitro fertilization/intracytoplasmic sperm injection (IVF/ICSI) treatment cycles due to several ovulation induction failures and/or IUI cycles were evaluated in the study period. The PCOS diagnosis was defined according to the Rotterdam criteria which included the presence of two of the following items (anovulation, hyperandrogenism and polycystic ovaries) (14). The exclusion criteria were as follows: uterine abnormality, history of unilateral oophorectomy, abnormal karyotype, recurrent implantation failures, severe male factor or azoospermia diagnosis, and other medical conditions that contraindicated ART and/or pregnancy.

The PCO phenotypes were defined as follows: phenotype A: oligo-ovulation or anovulation + hyperandrogenism + polycystic ovaries, phenotype B: oligo-ovulation or anovulation + hyperandrogenism; phenotype C: hyperandrogenism + polycystic ovaries; phenotype D: oligo-ovulation or anovulation + polycystic ovaries. The patients were evaluated regarding MetS prior to IVF/ICSI cycles for diagnosis. MetS was detected according to the National Cholesterol Education Program/Adult Treatment Panel III with the presence of at least three or more of the following clinical criteria: (1) waist circumference (WC) WC ≥ 88 cm; (2) triglyceride (TG) ≥ 150 mg/dL; (3) high-density lipoprotein-cholesterol (HDL-C) < 50 mg/dL; (4) blood pressure ≥ 130/85 mm Hg; and (5) fasting plasma glucose ≥ 110 mg/dL and (5) specific medication to any of these conditions.

Demographic information, medical history, history of obstetrics, current infertility history, and PCO symptoms are then recorded in a checklist. Weight and height, waist circumference (the midpoint between the lowest rib and the iliac crest), and systolic and diastolic blood pressure are measured. In addition, after 12 hours of fasting, serum lipid and glucose profiles as well as a 75 g oral glucose tolerance test, free testosterone, C-reactive protein (CRP), and TSH, were assessed.

All the participants underwent IVF/ICSI cycles with a flexible GnRH antagonist ovarian stimulation protocol. The controlled ovarian stimulation was initiated with gonadotropins recombinant follicle stimulation hormone (rFSH: Gonal-F^®^: Serono Laboratories Ltd, Geneva, Switzerland) on day 2 or 3 of the menstrual cycle. The GnRH-ant (Cetrotide^®^: 0.25 mg daily, cetrorelix acetate, Serono, Inc) was started when the leading follicle diameter was ≥ 13 mm, and then it continues until the administration of the oocyte trigger. The trans-vaginal monitoring sonography was started 5-7 days after the onset of ovarian stimulation, then every 2 or 3 days to adjust the dose of gonadotropin. The number and size of follicles were checked in each monitoring. When at least 2 follicles in the size of 17-18 mm were observed, the final oocyte triggering was performed by injection of 0.2 GnRH analog (Decapeptyl^®^: Ferring GmbH) ampoules and then 34-36 hours later the ovum pickup was planned. IVF/ICSI procedure was performed following the standard clinical technique. We planned the all-freeze strategy for all of the study participants be able to evaluate the results better. In summary, the embryo morphology was assessed according to Cummins and cols.’s (15) criteria by detecting the number and regularity of blastomeres and the degree of fragmentation on the third day. All top-quality embryos were frozen by the vitrification method three days after ovum pick-up. After two months frozen embryo transfer (FET) cycle was planned. All the patients received hormone replacement therapy with a down-regulated GnRH agonist for endometrial preparation. The endometrial preparation was started using 4 mg estradiol valerate daily if the endometrial thickness was less than 5 mm and serum estradiol level < 50 pg/mL. After 10-12 days of estradiol administration, if the favourable thickness of the endometrium (≥7 mm) was confirmed by ultrasound, estradiol valerate was continued with the same dose and then vaginal progesterone suppository (Cyclogest^®^ 400 mg twice a day) was administrated for luteal phase support. The number of transferred embryos was established on maternal age and embryo morphology, then one or maximally two cleavage stage embryos were transferred.

The height, weight, waist, and buttocks circumferences, and blood pressure were measured according to a standard protocol. Due to conducting these tests in two separate centers, the personnel of each center measured 10 samples together, and then an intra-class correlation coefficient was used to check the agreement. All blood samples were referred to a single laboratory center and all of the measurements were performed with the specific kits. The oocytes and embryo morphology were also assessed according to the standard guideline and to probe the agreement between two embryologists in the two centers, embryologists were requested to examine 10 identical specimens and then the kappa correlation coefficient was determined between the two observations.

### Outcome measures

The primary outcome was the relationship between MetS diagnosis and the number of MII oocytes (as an indicator of oocyte quality) and also the relationship between leading MetS diagnostic parameters (WC and BMI) and the number of MII oocytes. The secondary endpoints were the relationship between MetS diagnosis and FET cycle outcomes (embryo quality, fertilization and clinical pregnancy rates, as well as miscarriage, live birth, and obstetrics complication).

### Statistical analysis

The sample size was estimated on the basis of the standard deviations of the total number of retrieved oocytes in the MetS and non-MetS groups in the Madani and cols., study (16). Due to the small number of patients with MetS, the ratio of patients without MetS to those without MetS was considered to be 2:1 when calculating the sample size. Thus, by using specific formula the number of subjects 60 and 120 respectively in the MetS and non-MetS groups were required at a significance level (alpha level) of 0.05 and a power of 80%. Statistical analysis was performed using the Statistical Package for Social Sciences (SPSS Inc., Chicago, IL, USA) version 23.0. The chi-square test was used to the categorical variables between two groups and the results were presented as numbers/percentages. The Kolmogorov-Smirnov test was applied to detect the normality of continuous variables. The continuous variables with normal distribution were compared between groups by student’s t-test and reported as mean ± standard deviation (SD).

## RESULTS

Overall, 68 eligible infertile PCOS patients with MetS diagnosis and 126 without MetS participated in the present study. In the initial assessment, it was identified that the majority of the MetS patients (55.9%) had three diagnostic criteria: central obesity and elevated TG, and decreased serum HDL levels. The baseline characteristics of patients in two groups were compared in Table 1 and according to the results, there was no statistically significant difference between the two groups in terms of age, duration of infertility and type of infertility, PCOS phenotypes, etc. The diagnostic parameters of MetS such as BMI, serum triglycerides, HDL, FBS, GTT (2h), blood pressure, and waist circumference had significant differences between groups (P < 0.001 for all). The frequency of each diagnostic component of MetS included central obesity, hypertension, elevated TG and FBS levels, and decreased HDL levels were respectively 95.6%, 20.6%, 82.4%, 26.5%, and 95.6%.

**Table 1:** Comparison of the study population characteristics between PCOS women with and without MetS

Variables\Study group			MetS (n = 68)	Non-MetS (n = 126)	P-value
Women age (years)			31.8 ± 5.6	30.3 ± 6.1	0.09
Body mass index (kg/m^2^)			29.6 ± 4.4	26.0 ± 4.6	**<0.001**
Waist circumference, (cm)			93.9 ± 8.0	80.2 ± 7.8	**<0.001**
Central obesity (WC ≥ 88 cm), n (%)			65 (95.6)	41 (32.2)	**<0.001**
Systolic blood pressure (mmHg), (rang)			113.6 ± 14.5, (80-160)	107.3 ± 10.4, (85-140)	**0.001**
Diastolic blood pressure (mmHg), (rang)			73.3 ± 11.2, (50-100)	67.2 ± 8.6, (50-90)	**<0.001**
Blood pressure ≥ 130/85 (mmHg), n (%)			14 (20.6)	3 (2.4)	**<0.001**
Basal serum LH level, (IU/L)			7.3 ± 3.6	9.5 ± 12.5	0.15
Serum AMH level, (ng/mL)			8.8 ± 5.3	9.3 ± 5.2	0.6
LH/FSH ratio			1.4 ± 0.89	1.8 ± 1.87	0.1
Serum TSH level,)mIU/L)			2.1 ± 0.8	2.0 ± 0.9	0.3
Serum free testosterone			2.1 ± 2.0	1.6 ± 1.6	0.2
Serum CRP (positive), n (%)			7 (10.3)	11 (8.7)	0.7
Fasting Blood Sugar (mg/dL), (rang)			96.9 ± 11.4, (66-115)	91.7 ± 8.9, (73-112)	**<0.001**
FBS ≥ 110 mg/dL			18 (26.5)	6 (25.0)	**<0.001**
Serum fasting insulin (mIU/L)			12.1 ± 6.0	9.9 ± 6.5	0.1
Glucose tolerance test (2h), (mg/dL)			146.5 ± 54.7	110.6 ± 27.2	**<0.001**
Serum triglycerides (mg/dL), (rang)			199.7 ± 73.1, (76-383)	101.6 ± 33.1, (40-219)	**<0.001**
TG ≥ 150 (mg/dL), n (%)			56 (82.4)	6 (4.7)	**<0.001**
Serum HDL, (mg/dL), (rang)			40.7 ± 8.7, (27-91)	49.7 ± 11.0, (19-81)	**<0.001**
HDL < 50 mg/dL			62 (95.6)	65 (48.8)	**<0.001**
Cases under levothyroxine treatment			22 (32.4)	35 (27.6)	0.5
PCOS phenotype					0.6
	A		43 (63.2)	76 (60.3)	
	B		1 (1.5)	1 (0.8)	
	C		5 (7.4)	16 (12.7)	
	D		19 (27.9)	33 (26.2)	
Type of Infertility		Primary	23 (33.2)	51 (40.5)	0.5
		Secondary	45 (66.8)	95 (59.5)	

AMH: anti-müllerian hormone; CRP: C-reactive protein; FSH: follicle stimulation hormone; FBS: fasting blood sugar HDL: high-density lipoprotein; LH: luteinizing hormone; MetS: metabolic syndrome; PCOS: polycystic ovary syndrome’s: TG: triglycerides; TSH: thyroid stimulating hormone; WC: waist circumference. The PCO phenotypes were defined as follow: phenotype A: oligo-ovulation or anovulation + hyperandrogenism + polycystic ovaries, phenotype B: oligo-ovulation or anovulation + hyperandrogenism; phenotype C: hyperandrogenism + polycystic ovaries; phenotype D: oligo-ovulation or anovulation + polycystic ovaries. The statistically significant P values are in bold; the quantitative and qualitative variables are presented as mean (Standard Deviation) and number (percentage), respectively. Rang (minimum-maximum).

The relationship between BMI and the number of MII oocytes was evaluated with Pearson’s correlation coefficient test. A non-significant inverse relationship between BMI and the number of MII oocytes was found in the MetS group (r = -0.18, P-value = 0.13). Furthermore, there was no significant relationship between these parameters in the non-MetS group (r = -0.01, P-value = 0.84). There was no significant difference in terms of the mean number of MII oocytes between patients with and without central obesity (WC ≥ 88 cm) (12.7 ± 6.6 versus 13.2 ± 8.8, P = 0.6, respectively).

Table 2 shows the comparison of primary and secondary outcomes between the study groups. The analysis showed that the two groups had a statistically significant difference in terms of the ovarian stimulation duration and the total dose of used gonadotropins (P = 0.001). Although the total numbers of retrieved and MII oocytes in the MetS group were lower than the ones in the non-MetS group, the differences were not statistically significant. In a similar way, the fertilization rate and the numbers of obtained and top-quality embryos in the MetS group were lower in than the non-MetS group without statistically significant differences (P = 0.1, P = 0.2, and P = 0.08). There was no significant difference in the number of transferred embryos and endometrial thickness at ET day between groups. No multiple or twin pregnancy was reported in both groups. In the following, Despite the lower clinical pregnancy and higher miscarriage rates, and lower live birth rate in the MetS group in comparison to the non-MetS group, the differences were not statistically significant (P = 0.1). In terms of obstetrics complications, the MetS group was associated with a higher rate of GDM and preeclampsia in comparison to the non-MetS group; however, it was statistically significant only for preeclampsia rate (P = 0.02).

**Table 2:** Comparison of the IVF/ICSI and pregnancy outcomes between PCOS women with and without MetS

Variables\Study group	MetS (n = 68)	Non-MetS (n = 126)	P-value*
Duration of stimulation (days)	11.7 ± 2.0	10.0 ± 2.0	0.001
Total ampoule of used gonadotropins (75 IU)	27.2 ± 7.8	23.2 ± 6.1	0.001
Serum estradiol on oocytes triggering day	2388 ± 2003	2781 ± 2262	0.4
No. of retrieved oocytes	17.8 ± 9.8	18.0 ± 10.9	0.8
No. of metaphase II oocytes	13.0 ± 8.6	13.0 ± 7.9	0.9
Fertilization rate	0.80 ± 0.17	0.84 ± 0.16	0.1
No. of obtained embryo	8.6 ± 5.1	9.6 ± 6.3	0.2
No. of top quality embryos	6.8 ± 4.2	7.4 ± 6.0	0.08
aOHSS at risk rate; n (%)	40 (58.8)	78 ( 61.9)	0.7
Endometrial thickness at ET day (mm)	9.5 ± 1.5	9.4 ± 1.9	0.8
Chemical pregnancy rate per ET (%)	30/68 (44.1)	61/126 (48)	0.6
Clinical pregnancy rate per ET (%)	26/68 (38.2)	53/126 (41.7)	0.6
Miscarriage rate/per ET (%)	9/68 (13.2)	10/126 (7.6)	0.1
Live birth rate/per ET (%)	17/68 (25)	43/126 (34.1)	0.1
GDM/per clinical pregnancy (%)	6/26 (23)	8/53 (15)	0.3
Preeclampsia/per clinical pregnancy (%)	7/26 (27)	4/53 (7.5)	0.02
Preterm/per clinical pregnancy (%)	7/26 (27)	17/53 (32)	0.6

* tatistically significant *P* values < 0.05; the quantitative variables are presented as mean (Standard deviation).

a Risk of mild ovarian hyperstimulation syndrome.

## DISCUSSION

The present study findings revealed that PCOS women with MetS had higher gonadotropin requirements and duration of stimulation for COS. Moreover, MetS diagnosis was associated with a fewer number of MII oocytes, and fewer obtained top-quality embryos after IVF/ICSI cycles, however, the differences were not statistically significant. In the following, no significant effect of MetS was found on clinical pregnancy and live birth rates after IVF/ICSI/FET cycles. Regarding pregnancy complications, we found a significantly increased risk of preeclampsia in PCOS women with MetS diagnosis.

Recently, Lim and cols., in a systematic review and meta-analysis concluded that the risk of MetS was increased in women with PCOS and it was associated with obesity and metabolic features but not with markers of hyperandrogenism (8). Central obesity and IR are major risk factors for this disorder and many metabolic abnormalities of the MetS overlap with PCO (10). There is evidence to suggest that central obesity in these patients had deleterious effects on the reproductive outcome by inducing local and systemic oxidative stress (17). It was reported that IR, obesity, and dyslipidemia, which is a pathophysiological factor of MetS, have negative effects on pregnancy, fetal growth, and endometrial receptivity disorders (13). Furthermore, in a review article Cardozo and cols. reported negative effects of MetS on oocyte quality and reproductive outcomes (10). MetS are associated with chronic inflammation, which together with dyslipidemia may play a role in pregnancy disorders (18,19). Abnormal lipid metabolism causes endothelial destruction and thus affects placental perfusion (19). On the other hand, changes in carbohydrate metabolism due to IR and excessive carbohydrate intake may be associated with ovulation impairment and also affect endometrial growth and receptivity (20). Moreover, Bañuls and cols. showed that PCO patients with MetS diagnosis have increased lipolysis in the follicular fluid (21) and concluded that this altered metabolic status increased ROS production, ER stress, and leukocyte-endothelium interactions in PCOS with MetS diagnosis, all of which are related to vascular complications.

To our knowledge, few studies have evaluated the effect of MetS on fertility in PCOS women. He and cols. in the secondary analysis of a multicenter randomized trial in 1,508 women with PCOS indicated that patients with MetS required significantly higher and longer doses of gonadotropin along with the lower peak of estradiol level, fewer retrieved oocytes, available embryos, and a lower oocyte utilization rate than those with non-MetS. In agreement with He and cols. study, it was found that the duration of ovarian stimulation and total dose of used gonadotropins were significantly higher in women with MetS, which could be related to central obesity in these patients. However, no significant relationship was found between MetS diagnosis and other COS outcomes including the total number of retrieved and MII oocytes, obtained embryos, fertilization rate as well as clinical pregnancy, miscarriage and live birth rates. Although their study had a significantly higher miscarriage rate in the MetS group in their study. However, the live birth rate was not significantly different from that in the control group, consistent with our study (13). Comparing the severity of the diagnostic components of MetS indicated that the rate of impaired glucose metabolism in the present study was 26.5% versus 62.0% in He and cols.’s study; therefore, the discrepancy in the results of studies may be due to differences in the severity of the components of the MetS. Of course, the confounding factors affecting the rates of clinical pregnancy, miscarriage, and live birth are numerous and so no definite comment can be made in this regard. It is suggested that a study with a larger sample size be performed specifically to examine the effect of MetS on pregnancy outcomes following ART cycles.

Elsewhere, Li and cols. evaluated the effect of central obesity (WC ≥ 88 cm) on parameters of COS and laboratory, and pregnancy outcomes in PCOS patients in a retrospective case-control study (17). Their findings showed that patients with central obesity had significantly increased endocrine and metabolic disorders and needed a significantly higher dose of gonadotropins, and a longer duration of ovarian stimulation, but had significantly lower peak serum estradiol levels and fewer oocytes retrieved as well as lower implantation and live birth and rates. These studies suggest that changes in the maternal metabolic environment lead to abnormal changes in follicular fluid, which in turn reduces the quality of the oocytes and embryos. Robker and cols. concluded that obese women exhibit an altered ovarian follicular environment, especially elevated metabolite, CRP, and androgen activity levels, which may be associated with poorer reproductive outcomes in an observational study (22). Elevated CRP level in the follicular fluid of obese women is particularly important because it may indicate inflammation and increased oxidative stress, which is associated with the decreased potential of oocyte growth (21,22). Increased oxidative stress may be a mechanism associated with obesity which affects the oocyte quality. In the present study, no significant difference in the ratio of patients with serum positive CRP was found between MetS and non-MetS patients.

Furthermore, in the present study, the risk of preeclampsia was increased in patients with MetS. The endothelial damage due to abnormal lipid metabolism may reduce placental perfusion and lead to preeclampsia or spontaneous preterm birth (13,19). Grieger and cols., in a prospective study, evaluated the relationship between MetS and adverse pregnancy outcomes in the non-ART population (23). A total number of 5,530 women were included, 12.3% of which were diagnosed with MetS (n = 684). The risk of preeclampsia in women with MetS was increased by a factor of 1.63 (95% CI 1.23 to 2.15) as well as the risk of GDM by 3.71 (95% CI 2.42 to 5.67) (23). They concluded that more than half of the women who had MetS in early pregnancy developed a pregnancy complication compared with just over a third of women without MetS. Moreover, while increasing BMI increases the probability of GDM, accompanying MetS exacerbates this risk and more studies are warranted to clarify if individual MetS components act synergistically or independently (23). Recently, Baldini and cols. in a review article concluded that when MetS and obesity are found in ART patients, they pose a significantly increased risk of mobility and mortality, therefore these patients should be more accurately supervised through a multidisciplinary approach (24).

A recurrent study in the Iranian population reported that the prevalence of MetS in infertile PCOS patients was 19.7% according to ATP III criteria and Rotterdam criteria, in their study the hormonal, anthropometric parameters, as well as glucose and lipid profiles, were compared between MetS and non-MetS women with PCOS diagnosis but the fertility or IVF/ICSI cycles outcome was not evaluated (16). The follow-up of the patients until birth and comparison of pregnancy complications between groups were the strengths of the present study. The low sample size was the study limitation; however, it was due to the time and financial constraints of the prospective study. Although all patients diagnosed with MetS were examined in two infertility centers for a year, a multi-center study with a higher sample size is needed for definitive conclusions in this field.

In summary, MetS diagnosis in PCOS patients was associated with the increased requirement of gonadotropins and the duration of COS. Despite the lower number of MII oocytes and top-quality embryos followed by lower clinical pregnancy and live birth rates after FET cycles in MetS patients, the differences were not statistically significant. Nevertheless, this amount of discrepancy, which is not statistically significant, can be clinically important for clinicians; therefore further studies with larger sample sizes are recommended to clarify the risk of MetS in patients undergoing the ART cycle. Among the obstetrics complications, the rate of preeclampsia was significantly higher in patients with MetS diagnosis, so it is suggested that prenatal care be provided for these patients as a high-risk group.
